# Association of the ROX index with mortality in sepsis patients: a retrospective study

**DOI:** 10.3389/fmed.2025.1709669

**Published:** 2025-12-02

**Authors:** Jiali Wu, Jing Zhao, Xiaojing Ji, Xiangfei Kang, Bo Li, Jinyuan Zhu

**Affiliations:** 1 Department of Emergency, General Hospital of Ningxia Medical University, Yinchuan, China; 2 Department of Clinical Laboratory, Ningdong Center for Disease Control and Prevention, Yinchuan, China; 3 Department of Critical Care Medicine, General Hospital of Ningxia Medical University, Yinchuan, China

**Keywords:** ROX index, sepsis, mortality, MIMIC-IV, clinical outcomes

## Abstract

**Background:**

This study aims to explore the association between the ROX index and clinical outcomes in patients with sepsis.

**Methods:**

Data were extracted from the Medical Information Mart for Intensive Care IV (MIMIC-IV) database, including adult sepsis patients admitted to the intensive care unit (ICU). The primary outcome was 28-day mortality, while secondary outcomes included 7-day and 14-day mortality, ICU mortality, ICU length of stay (LOS), and hospital LOS. Restricted cubic spline models and Cox regression models were used to assess the associations between the ROX Index and clinical outcomes.

**Results:**

A total of 23,502 sepsis patients were included, who were stratified into high (ROX ≥ 6.46) and low ROX (ROX < 6.46) strata using a data-derived threshold survival analysis. In the unadjusted model, the high ROX stratum exhibited a significantly lower risk of 28-day mortality (HR = 0.33, 95% CI: 0.31–0.35, *P* < 0.001), with consistent findings after adjustment for age, sex, and SOFA score respectively. Similar trends were observed for ICU, 7-day, and 14-day mortality. Restricted cubic spline analysis revealed a nonlinear “L”-shaped association, with the 28-day mortality risk decreasing until reaching a plateau at a ROX index of approximately 10.50. Additionally, the shortest ICU and hospital LOS were observed at ROX thresholds of 9.56 (4.87 days) and 9.29 (12.05 days), respectively. The ROX index showed a moderate predictive accuracy for 28-day mortality (AUC = 0.63, 95% CI: 0.62–0.64), outperforming the SOFA score (AUC = 0.59, 95% CI: 0.58–0.60). Subgroup analyses confirmed consistent associations across demographic and clinical subgroups (overall HR = 0.33, 95% CI: 0.31–0.35), with significant interaction effects observed in gender, patients with septic shock, heart failure, diabetes, chronic lung disease, and those not receiving non-invasive ventilation (all *P* < 0.05).

**Conclusion:**

In patients with sepsis, a higher ROX index is associated with significantly lower mortality rates and shorter ICU and hospital stays. However, the ROX index demonstrated moderate predictive accuracy.

## Introduction

Sepsis is a life-threatening organ dysfunction caused by a dysregulated host response to an infection that can originate anywhere in the body (1), with a high mortality rate. While the treatment methods for sepsis are constantly advancing, its mortality remains high. Early prediction of sepsis-related mortality is crucial for the timely initiation of targeted interventions that can potentially improve patient outcomes (2, 3). However, there are numerous tools available for the assessment of sepsis prognosis, but no optimal tool currently exists that can accurately forecast the risk of death due to sepsis (4). Different indicators vary significantly in terms of accuracy, sensitivity, and specificity in prognosis assessment, and each has its own advantages and disadvantages in clinical application.

The Respiratory Oxygenation Index (ROX) is a valuable predictor used to assess the efficiency of oxygenation in critical patients, particularly those receiving non-invasive respiratory support such as high-flow nasal cannula (HFNC) treatment or non-invasive ventilation (NIV) (5, 6). It provides a rapid and non-invasive method to evaluate the response to respiratory support that can predict the need for escalation of care, including intubation and mechanical ventilation, and it can also help identify patients at low or high risk for intubation (7). Given that the ROX index has demonstrated good performance in the assessment of patients with respiratory failure, there has been an increasing number of studies on its application in other diseases, such as acute respiratory distress syndrome and COVID-19.

Patients with sepsis often develop concurrent respiratory dysfunction (8), and the severity of this dysfunction is associated with patient mortality. Given the role of the ROX index in evaluating the severity of illness in patients with respiratory failure, the ROX index may be one of the potentially useful indicators for assessing the prognosis of septic patients. However, this relationship has not been thoroughly explored in existing research. Therefore, this study aims to investigate the relationship between the ROX index and sepsis mortality and to evaluate its potential as an effective predictor of sepsis outcomes. In order to provide a useful method for prognosis assessment in sepsis patients.

## Methods

### Study design and data source

This is a retrospective cohort study, we employed data extracted from the Medical Information Mart for Intensive Care IV (MIMIC-IV, version 3.0) database, a large deidentified dataset of patients admitted to the intensive care unit of the Beth Israel Deaconess Medical Center (BIDMC) in Boston, contains data for over 65,000 patients information (9, 10). To ensure appropriate use of this resource, we completed the required training courses and obtained certification (Certification number: 54441907). The Institutional Review Board of BIDMC granted a waiver of informed consent and approved the sharing of the research resource. Therefore, for this manuscript, both the statement of ethical approval and the requirement for informed consent were waived. Throughout the study, we adhered to the ethical requirements and guidelines for using the data, including compliance with the Declaration of Helsinki.

### Study participant selection

**Figure 1:**
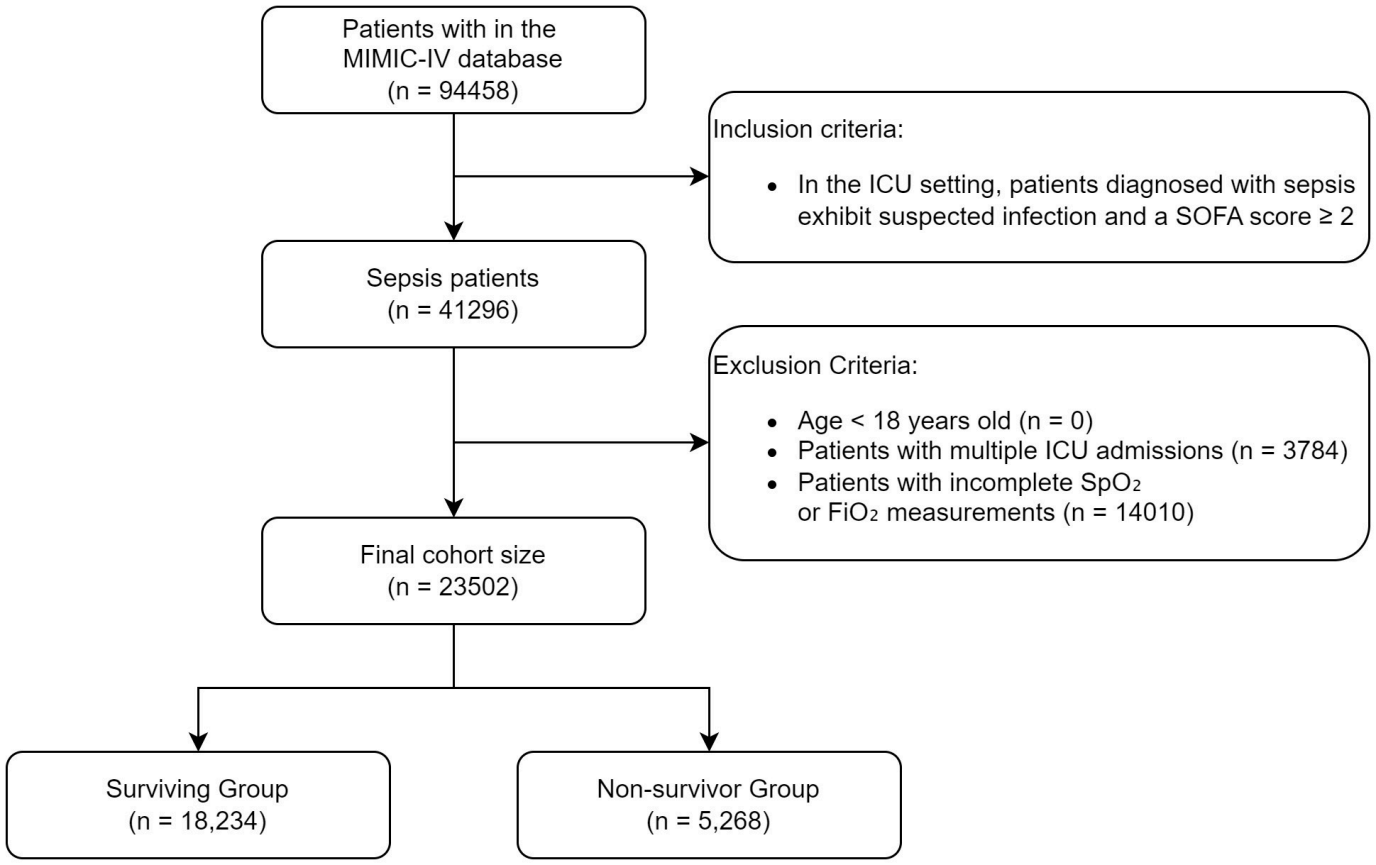
Flowchart of participants' enrollment from MIMIC-IV.

In this study, we included all patients aged 18 years or older with a diagnosis of sepsis, as defined by the Sepsis-3 criteria (11), who were admitted to the ICU and recorded in the MIMIC-IV database. The following exclusion criteria were applied: patients with multiple ICU admissions during the study period, and individuals for whom SpO_2_ or FiO_2_ data were unavailable at the time of ICU admission. A flowchart illustrating the enrollment process of participants is shown in Figure 1.

### Data extraction

For data extraction from the MIMIC-IV database, we employed Structured Query Language (SQL) to retrieve comprehensive clinical characteristics of the patients. The extracted data encompassed demographic information, including age, sex, and weight, as well as comorbidities such as hypertension, congestive heart failure, diabetes mellitus, and chronic pulmonary disease. Additionally, we obtained the mean values of FiO_2_ (fraction of inspired oxygen) and PaO_2_ (partial pressure of arterial oxygen) on the first day of admission, along with the maximum respiratory rate, minimum mean arterial pressure, maximum white blood cell count, minimum hemoglobin and platelet counts, maximum blood urea nitrogen, creatinine, potassium, sodium, chloride, calcium levels, maximum prothrombin time (PT), maximum international normalized ratio (INR), and maximum lactate level. Furthermore, the Charlson Comorbidity Index (CCI), Sequential Organ Failure Assessment (SOFA) score, and Simplified Acute Physiology Score (SAPS) II were also calculated using data collected within the first 24 h of ICU admission.

### Outcomes

The primary outcome was 28-day mortality. Secondary outcomes included 7-day and 14-day mortality, ICU mortality, length of hospital stay (LOS) and ICU Length of Stay (ICU LOS). The date of death recorded in the MIMIC-IV database was derived from hospital records and state records.

### Statistical analysis

Statistical analysis was performed using the R software (version 4.4.0). Missing variables were imputed using the random forest algorithm. This method can handle both numerical and categorical missing values by constructing predictive models to estimate missing data, with multiple imputations performed to ensure the robustness of the results. The imputed datasets were used for subsequent statistical analyses. To assess the relationship between the ROX Index and 28-day survival, we used the surv_cutpoint function from the survminer package to determine the optimal cut-off point. Subsequently, patients were stratified into two strata: the high ROX stratum and the low ROX stratum.

Continuous variables following a normal distribution are presented as mean ± standard deviation (SD) and compared using Student's *t*-test. For non-normally distributed continuous variables, the median and interquartile range (IQR) were reported, and comparisons were made using the Mann–Whitney *U*-test. Categorical variables are described as frequencies and percentages and analyzed using the chi-squared test. A *P* value < 0.05 was considered statistically significant.

To investigate the association between the ROX Index and patient outcomes, we constructed multivariable Cox proportional hazards regression models, taking into account potential confounders that could influence the results. Based on univariate analysis, relevant guidelines, and clinical expert opinions, four models with different covariates were established: model 1 adjusted for age; model 2 added gender to model 1; model 3 included the SOFA score in addition to model 2. We calculated the absolute risk differences (ADR) across models to quantify the improvement in risk prediction accuracy at clinically important thresholds. To assess model calibration, we developed calibration curves evaluating the ROX index for predicting mortality in septic patients.

Kaplan–Meier survival curves were constructed to assess the impact of the ROX Index on survival outcomes, and restricted cubic spline (RCS) curves were constructed to examine the associations between the ROX Index and mortality risk, as well as the length of hospital and ICU stay. The area under the receiver operating characteristic (ROC) curve was used to assess the predictive ability of mortality. Subgroup analyses using multivariable Cox proportional hazards models were also performed to assess the consistency and stability of the ROX Index's predictive power across different subgroups, adjusting for potential confounders within each subgroup, with the results summarized in a forest plot.

To evaluate the robustness of the relationship between the ROX Index and the prognosis of septic patients, we conducted several sensitivity analyses. Firstly, the ROX Index was incorporated as a continuous variable into the Cox proportional hazards model to assess its direct impact on prognosis. Secondly, the ROX Index was truncated at the median and divided into two strata, and the Cox model was applied again to compare the survival differences between patients in the two strata. Thirdly, to avoid bias caused by missing values, we deleted all cases with missing values and reconstructed the Cox model. Fourthly, all variables showing statistical significance in the univariate analysis were included in the Cox model to adjust for potential confounding factors. Finally, considering the possible impact of pulmonary infection, we excluded cases with concurrent pulmonary infection and re-established the Cox model to further evaluate the impact of the ROX Index on patients' prognosis.

## Results

### Baseline characteristics

In this study, a total of 23,502 patients were excluded. Among the final cohort, there were 18,234 survivors and 5,268 non-survivors. The ROX Index was significantly lower in patients 28-day non-survivor patients compared to surviving patients (Table 1).

**Table 1:** Baseline characteristics of patients with sepsis.

**Baseline characteristics**	**Surviving stratum (*n* = 18,234)**	**Non-survivor stratum (*n* = 5,268)**	***P* value**
Age, median (IQR), year	64.55 ± 15.70	70.44 ± 15.34	< 0.001
Weight, median (IQR), kg	80.80 (27.80)	76.30 (27.00)	< 0.001
15.6-2.2,-1.3498ptMale, *n* (%)	11,098 (60.86%)	3,018 (57.29%)	< 0.001
**Comorbidities**			
Hypertension, *n* (%)	9,034 (49.54%)	2,326 (44.15%)	< 0.001
15.6-2.2,-1.3498ptSeptic shock, *n* (%)	2,801 (15.36%)	2,061 (39.12%)	< 0.001
**History of disease**			
Heart failure comorbidity, *n* (%)	5,524 (30.30%)	2,001 (37.98%)	< 0.001
Diabetes, *n* (%)	5,744 (31.50%)	1,678 (31.85%)	0.641
Chronic lung disease, *n* (%)	5,192 (28.47%)	1,636 (31.06%)	< 0.001
CCI	5.00 (4.00)	6.00 (4.00)	< 0.001
SOFA score, median (IQR)	3.00 (2.00)	4.00 (3.00)	< 0.001
15.6-2.2,-1.3498ptSAPS II score, median (IQR)	37.00 (17.00)	50.00 (22.00)	< 0.001
**Treatments after ICU admission**			
HFNC, *n* (%)	1,120 (6.14%)	433 (8.22%)	< 0.001
Non-invasive ventilation, *n* (%)	867 (4.75%)	188 (3.57%)	< 0.001
15.6-2.2,-1.3498ptInvasive ventilation, *n* (%)	13,025 (71.43%)	4,209 (79.90%)	< 0.001
**Vital signs at ICU admission**			
Heart rate, median (IQR), beats/min	103.00 (26.00)	111.00 (32.00)	< 0.001
Respiratory rate, median (IQR), bpm	27.00 (8.00)	30.00 (9.00)	< 0.001
Mean blood pressure, median (IQR), mmHg	58.00 (12.00)	54.00 (17.00)	< 0.001
SpO_2_, median (IQR), (%)	93.00 (5.00)	91.00 (8.00)	< 0.001
15.6-2.2,-1.3498ptROX index, median (IQR)	9.98 (4.37)	8.31 (5.40)	< 0.001
**Laboratory tests**			
WBC count, median (IQR), × 10^9^/L	14.20 (8.40)	15.45 (10.60)	< 0.001
Hemoglobin, median (IQR), g/L	9.60 (2.90)	9.40 (3.20)	< 0.001
Platelet count, median (IQR), × 10^9^/L	159.00 (109.00)	160.00 (135.00)	0.005
BUN, median (IQR), mg/dl	21.00 (19.00)	34.00 (31.00)	< 0.001
Creatinine, median (IQR), mg/dl	1.10 (0.80)	1.60 (1.70)	< 0.001
Potassium, median (IQR)	4.50 (0.80)	4.70 (1.20)	< 0.001
Sodium, median (IQR), mmol/L	140.00 (5.00)	141.00 (7.00)	< 0.001
Chloride, median (IQR)	107.00 (8.00)	106.00 (10.00)	< 0.001
Calcium, median (IQR)	8.43 (0.80)	8.60 (1.00)	< 0.001
PT, median (IQR)	15.00 (4.30)	16.70 (10.30)	< 0.001
INR, median (IQR)	1.38 (0.40)	1.50 (1.00)	< 0.001
15.6-2.2,-1.3498ptLactate, median (IQR)	2.30 (1.75)	3.10 (4.40)	< 0.001
**LOS**			
Length of ICU stay, median (IQR), day	3.63 (5.89)	4.27 (6.54)	0.002
Length of hospital stay, median (IQR), day	10.20 (12.77)	6.65 (9.87)	< 0.001

All indicators were based on the first 24 h of ICU admission. We selected the maximum values for heart rate, respiratory rate, WBC, BUN, creatinine, potassium, sodium, chloride, calcium, PT, INR, and lactate, while minimum values were recorded for MAP, SpO_2_, hemoglobin, and platelets. The ROX index was calculated using the daily average upon admission. CCI, Charlson comorbidity index; SOFA, sequential organ failure assessment; SAPS II, simplified acute physiology score II; HFNC, high-flow nasal cannula, MAP, mean arterial pressure; SpO_2_, peripheral oxygen saturation; ROX, respiratory oxygenation; WBC, white blood cell count; BUN, blood urea nitrogen; PT, prothrombin time; INR, international normalized ratio; IQR, interquartile range; SD, standard deviation.

In Table 2, we applied survival analysis methods to stratify the ROX Index into two strata: the low ROX stratum (ROX < 6.46, *n* = 3,910) and the high ROX stratum (ROX ≥ 6.46, *n* = 19,592). Patients in the low ROX stratum were younger, had higher body weight, a higher proportion of females, and a higher proportion of septic shock. They also had a higher prevalence of heart failure and chronic lung disease, higher SOFA and SAPS II scores, and a higher proportion of HFNC and non-invasive ventilation, higher heart rates and respiratory rates, and lower mean blood pressure and SpO_2_. In laboratory tests, patients in the low ROX stratum had higher White Blood Cell (WBC) count, hemoglobin, platelet count, Blood Urea Nitrogen (BUN), creatinine, potassium, sodium, calcium, PT, INR, and lactate levels compared to those in the high ROX stratum, while chloride levels were lower in the low ROX stratum than in the high ROX stratum. The total length of hospital stay and ICU stay was longer in the low ROX stratum (Table 2).

**Table 2:** Baseline characteristics of patients with sepsis stratify by ROX index.

**Baseline characteristics**	**Low ROX stratum (*n* = 2,767)**	**High ROX stratum (*n* = 20,735)**	***P* value**
Age, median (IQR), year	64.85 ± 16.63	66.08 ± 15.64	< 0.001
Weight, median (IQR), kg	81.20 (29.43)	80.00 (27.70)	< 0.001
15.6-2.2,-1.3498ptMale, *n* (%)	2,287 (58.49%)	11,829 (60.38%)	0.029
**Comorbidities**			
Hypertension, *n* (%)	1,714 (43.84%)	9,646 (49.23%)	< 0.001
15.6-2.2,-1.3498ptSeptic shock, *n* (%)	1,413 (36.14%)	3,449 (17.60%)	< 0.001
**History of disease**			
Heart Failure Comorbidity, *n* (%)	1,408 (36.01%)	6,117 (31.22%)	< 0.001
Diabetes, *n* (%)	1,218 (31.15%)	6,204 (31.67%)	0.539
Chronic lung disease, *n* (%)	1,197 (30.61%)	5,631 (28.74%)	0.020
CCI, median (IQR)	5.00 (4.00)	5.00 (4.00)	0.002
SOFA score, median (IQR)	4.00 (3.00)	3.00 (2.00)	< 0.001
15.6-2.2,-1.3498ptSAPS II score, median (IQR)	46.00 (25.00)	39.00 (18.00)	< 0.001
**Treatments after ICU admission**			
HFNC, *n* (%)	617 (15.78%)	936 (4.78%)	< 0.001
Non-invasive ventilation, *n* (%)	221 (5.65%)	834 (4.26%)	< 0.001
15.6-2.2,-1.3498ptInvasive ventilation, *n* (%)	2,673 (68.36%)	14,561 (74.32%)	< 0.001
**Vital signs at ICU admission**			
Heart rate, median (IQR), beats/min	115.00 (32.00)	103.00 (27.00)	< 0.001
Respiratory rate, median (IQR), bpm	33.00 (8.00)	27.00 (8.00)	< 0.001
Mean blood pressure, median (IQR), mmHg	55.00 (16.38)	58.00 (12.50)	< 0.001
SpO_2_, median (IQR), (%)	88.00 (7.00)	93.00 (5.00)	< 0.001
15.6-2.2,-1.3498ptROX index, median (IQR)	5.14 (1.52)	10.38 (3.98)	< 0.001
**Laboratory tests**			
WBC count, median (IQR), × 10^9^/L	15.20 (11.00)	14.30 (8.50)	< 0.001
Hemoglobin, median (IQR), g/L	9.90 (3.30)	9.50 (2.90)	< 0.001
Platelet count, median (IQR), × 10^9^/L	169.00 (137.00)	157.00 (111.00)	< 0.001
BUN, median (IQR), mg/dl	30.00 (29.00)	22.00 (21.00)	< 0.001
Creatinine, median (IQR), mg/dl	1.50 (1.50)	1.10 (0.90)	< 0.001
Potassium, median (IQR)	4.60 (1.10)	4.50 (0.80)	< 0.001
Sodium, median (IQR), mmol/L	140.00 (6.00)	140.00 (5.00)	< 0.001
Chloride, median (IQR)	105.00 (9.00)	107.00 (8.00)	< 0.001
Calcium, median (IQR)	8.50 (0.90)	8.50 (0.80)	0.081
PT, median (IQR)	16.10 (7.70)	15.10 (4.72)	< 0.001
INR, median (IQR)	1.50 (0.80)	1.40 (0.40)	< 0.001
15.6-2.2,-1.3498ptLactate, median (IQR)	2.71 (3.97)	2.39 (1.80)	< 0.001
**LOS**			
Length of ICU stay, median (IQR), day	4.78 (8.79)	3.64 (5.49)	< 0.001
Length of hospital stay, median (IQR), day	10.04 (15.33)	9.35 (11.03)	0.045

The ROX Index was stratified into two strata based on survival analysis: the low ROX stratum (ROX < 6.46) and the high ROX stratum (ROX ≥ 6.46). All indicators were based on the first 24 h of ICU admission. We selected the maximum values for heart rate, respiratory rate, WBC, BUN, creatinine, potassium, sodium, chloride, calcium, PT, INR, and lactate, while minimum values were recorded for MAP, SpO_2_, hemoglobin, and platelets. The ROX Index was calculated using the daily average upon admission. CCI, Charlson comorbidity index; SOFA, sequential organ failure assessment; SAPS II, simplified acute physiology score II; HFNC, high-flow nasal cannula; MAP, mean arterial pressure; SpO_2_, peripheral oxygen saturation; ROX, respiratory oxygenation; WBC, white blood cell count; BUN, blood urea nitrogen; PT, prothrombin time; INR, international normalized ratio; IQR, interquartile range; SD standard deviation.

### Relationship between ROX index and patient outcomes

**Figure 2:**
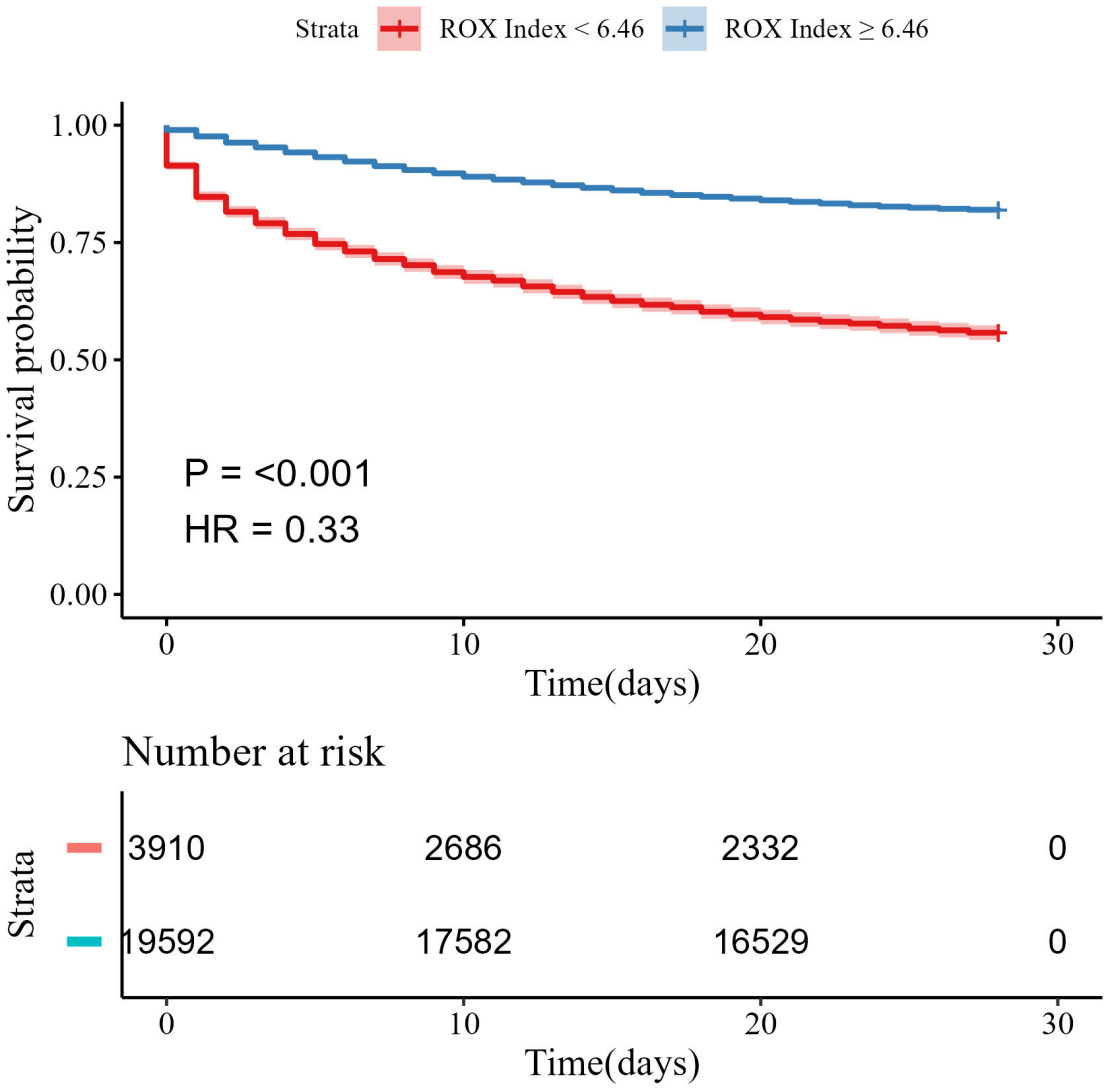
Kaplan–Meier analysis for survival probability in patients with sepsis.

In the multivariate analysis, we constructed four separate Cox regression models, adjusting for age, sex, and SOFA score respectively to evaluate the 7-day, 14-day, 28-day, and ICU mortality among patients. For the primary outcome of 28-day mortality, the unadjusted model indicated that patients with a high ROX Index had a significantly lower risk of death compared to those with a low ROX index, the HR of 28-day mortality was 0.33 (95% CI 0.31–0.35, *P* < 0.001). After further adjustments for age, sex, and SOFA score, respectively, the risk of death remained lower for patients in the high ROX stratum, and the HR of 28-day mortality was 0.31 (95% CI 0.29–0.33, *P* < 0.001), 0.31 (95% CI 0.30–0.33, *P* < 0.001), and 0.33 (95% CI 0.31–0.34, *P* < 0.001). Similarly, for ICU mortality, 7-day, and 14-day mortality, patients in the high ROX stratum also exhibited a lower risk of death than those in the low ROX stratum (Table 3). Table 4 shows the absolute risk differences (ARD) for mortality, derived from Cox regression models, between patients with low and high ROX indices. The low ROX stratum consistently showed a higher risk of death across all time points compared to the high ROX stratum. This increased risk persisted even after progressively adjusting for age, gender, and SOFA score, with ARDs ranging from 0.16 to 0.28 across the different models. Figure 2 shows the cumulative mortality, the risk of 28-day mortality was lower in the high ROX stratum compared to the low ROX stratum.

**Table 3:** Relationships between ROX index and mortality in patients with sepsis.

**Exposure**	**Crude model**		**Model 1**		**Model 2**		**Model 3**	
	**HR (95% CI)**	***P*** **value**	**HR (95% CI)**	***P*** **value**	**HR (95% CI)**	***P*** **value**	**HR (95% CI)**	***P*** **value**
ICU-mortality	0.39 (0.36–0.42)	< 0.001	0.37 (0.34–0.40)	< 0.001	0.37 (0.34–0.40)	< 0.001	0.39 (0.36–0.42)	< 0.001
28-day mortality	0.33 (0.31–0.35)	< 0.001	0.31 (0.29–0.33)	< 0.001	0.31 (0.30–0.33)	< 0.001	0.33 (0.31–0.34)	< 0.001
14-day mortality	0.30 (0.28–0.32)	< 0.001	0.29 (0.27–0.31)	< 0.001	0.29 (0.27–0.31)	< 0.001	0.30 (0.28–0.32)	< 0.001
7-day mortality	0.26 (0.24–0.28)	< 0.001	0.25 (0.23–0.27)	< 0.001	0.25 (0.23–0.27)	< 0.001	0.26 (0.24–0.28)	< 0.001

Model 1 adjusted for age; model 2 added gender to model 1; model 3 included SOFA score in addition to model 2.

**Table 4:** Absolute risk differences between ROX index and mortality in patients.

**Exposure**	**Crude model**	**Model 1**	**Model 2**	**Model 3**
	**ARD (95% CI)**	**ARD (95% CI)**	**ARD (95% CI)**	**ARD (95% CI)**
28-day mortality	0.27 (0.23–0.31)	0.28 (0.23–0.32)	0.27 (0.23–0.32)	0.26 (0.21–0.30)
14-day mortality	0.22 (0.21–0.24)	0.23 (0.21–0.24)	0.23 (0.21–0.24)	0.21 (0.20–0.23)
7-day mortality	0.16 (0.15–0.17)	0.17 (0.16–0.18)	0.17 (0.16–0.18)	0.16 (0.15–0.17)

Absolute risk differences (ARD) were calculated using multivariable adjustment: model 1 for age; model 2 for age and gender; model 3 for age, gender, and SOFA score.

**Figure 3:**
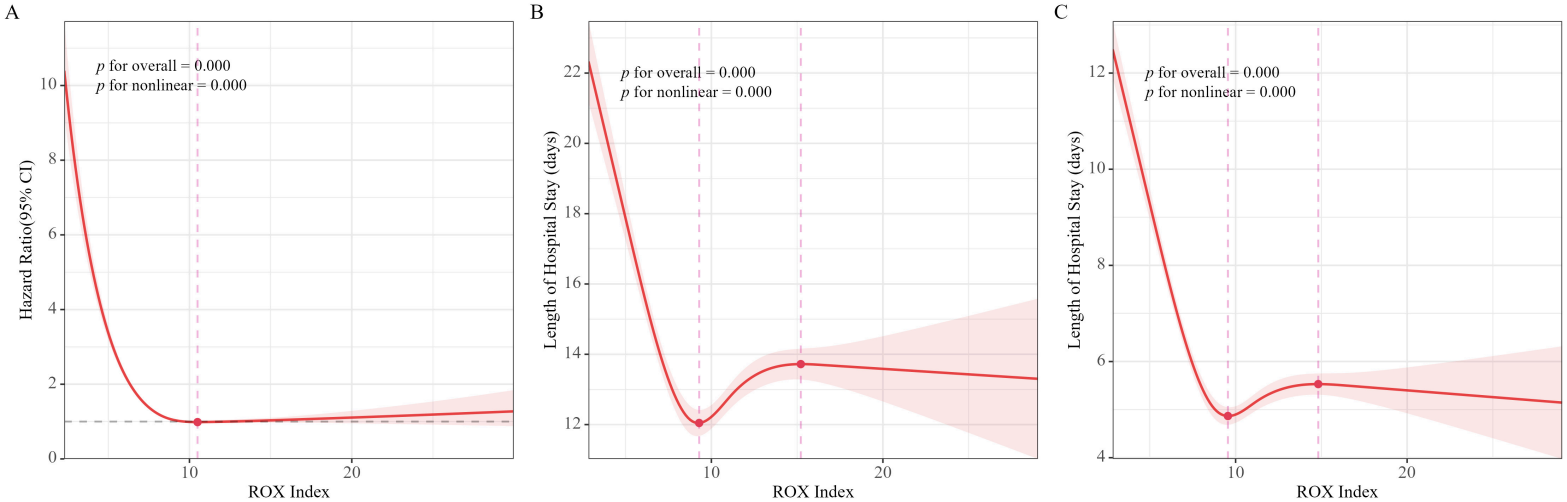
Restricted cubic spline curve of ROX index with mortality, length of hospital stay, and length of ICU stay in patients with sepsis. **(A)** RCS of ROX index with mortality, **(B)** RCS length of hospital stay, **(C)** RCS of length of ICU stay.

The RCS curve for the ROX index and 28-day mortality demonstrates a nonlinear, “L”-shaped relationship (Figure 3). As the ROX index increases, the 28-day mortality risk decreases. The risk reaches its lowest point at a ROX index of 10.50, after which, as the ROX index continues to increase, the mortality risk no longer declines significantly (Figure 3A). Similarly, both the LOS and ICU LOS decrease with increasing ROX index, and the RCS curve also demonstrating a nonlinear, “L”-shaped relationship. When the ROX index reaches 9.29 (Figure 3B) and 9.56 (Figure 3C), the hospital and ICU stays were minimized, at 12.05 and 4.87 days, respectively. Beyond these thresholds, additional increases in the ROX index do not result in notable changes in either hospital or ICU stay duration.

### Predictive performance of ROX index

**Figure 4:**
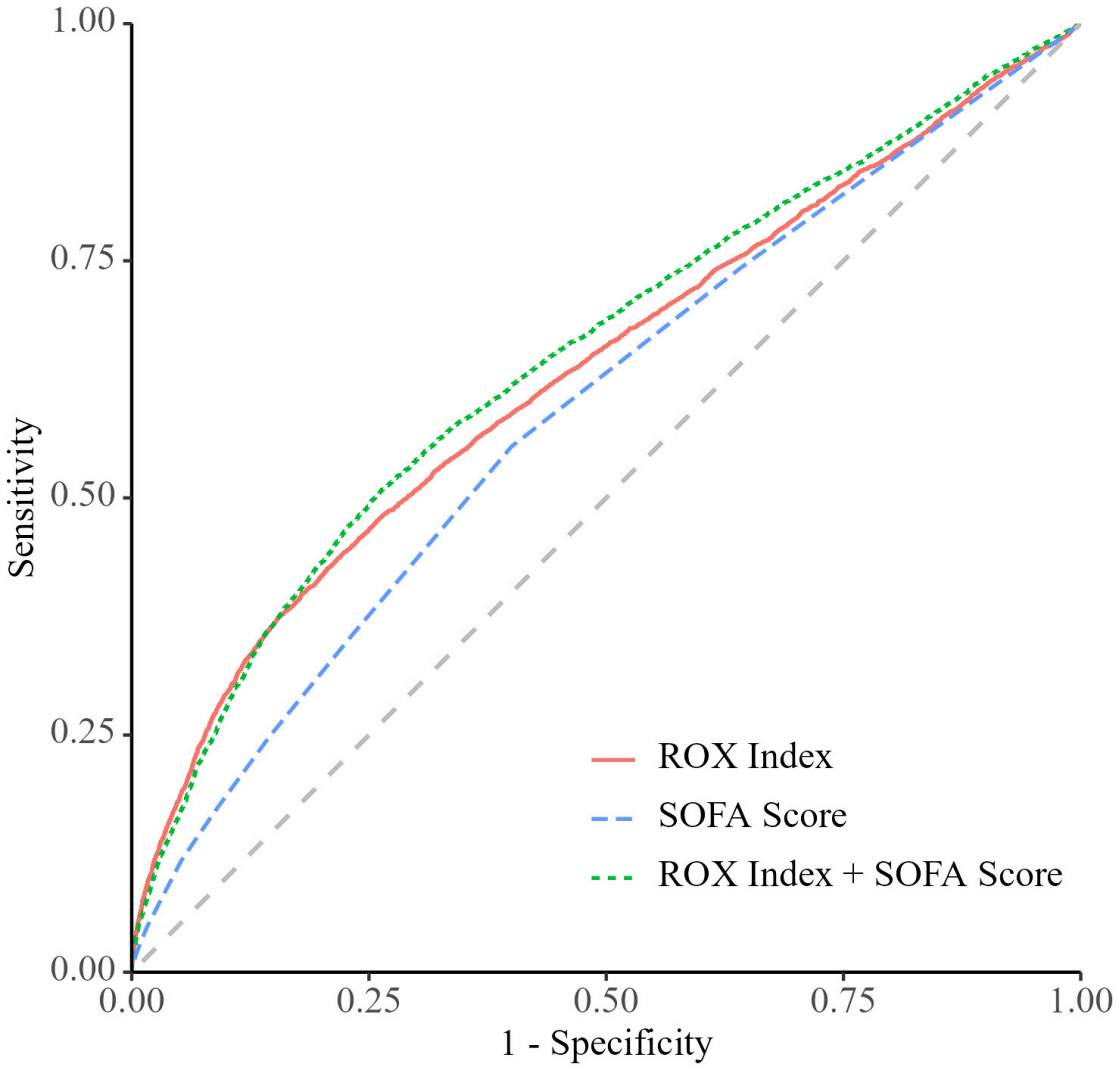
ROC analysis for the prediction of 28-day mortality compared to SOFA scores.

**Figure 5:**
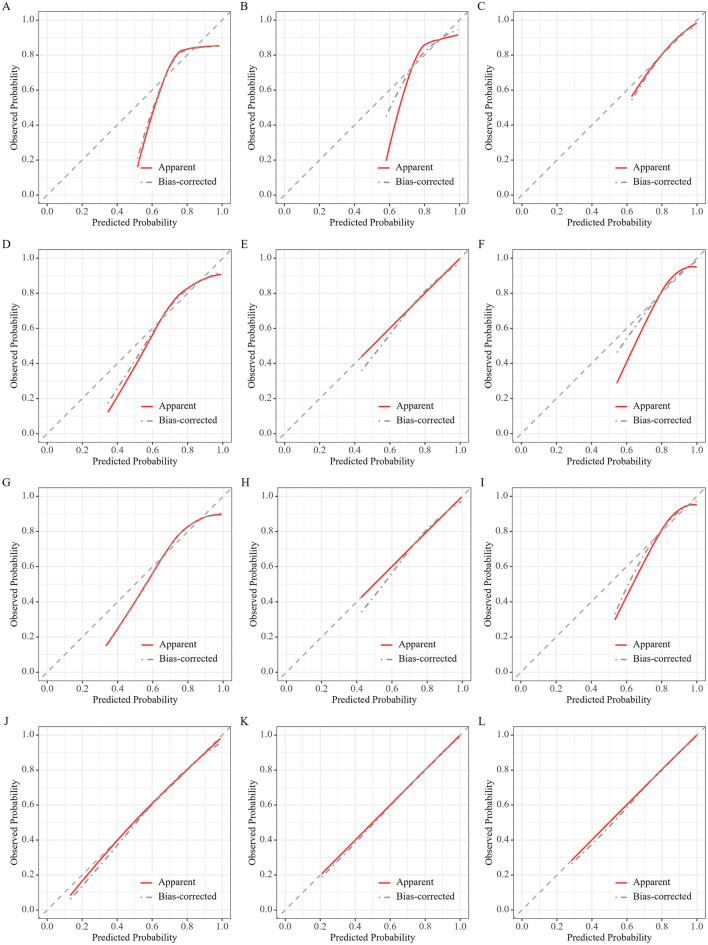
Calibration curves for the ROX index in predicting mortality. Plots show the model performance at 28 **(A, D, G, J)**, 14 **(B, E, H, K)**, and 7 **(C, F, I, L)** days for the unadjusted model **(A–C)**, the model adjusted for age **(D–F)**, the model adjusted for age and sex **(G–I)**, and the model adjusted for age, sex, and SOFA score **(J–L)**.

The area under the ROC (AUROC) curve of the ROX index for 28-day mortality in patients with sepsis was 0.63 (95% CI: 0.62–0.64), with a sensitivity of 0.42 (95% CI: 0.37–0.49) and specificity of 0.80 (95% CI: 0.74–0.85). Additionally, the AUROC of the ROX index was superior to that of the SOFA score, which had an AUC of 0.59 (95% CI: 0.59–0.59; Table 5 and Figure 4). Figure 5 showed the ROX index in predicting the risk of mortality in sepsis. Overall, there is good consistency between the predicted and observed probabilities.

**Table 5:** Predictive performance of ROX index and SOFA score for 28-day mortality.

**Variable**	**Cutoff value**	**Sensitivity (95% CI)**	**Specificity (95% CI)**	**Accuracy (95% CI)**	**AUC (95% CI)**
ROX index	6.90	0.37 (0.42–0.49)	0.74 (0.80–0.85)	0.68 (0.72–0.74)	0.62 (0.63–0.64)
SOFA score	3.50	0.54 (0.55–0.57)	0.59 (0.60–0.61)	0.59 (0.59–0.59)	0.58 (0.59–0.60)
ROX index + SOFA score	0.24	0.48 (0.50–0.56)	0.69 (0.74–0.77)	0.66 (0.69–0.70)	0.64 (0.65–0.66)

ROX, respiratory oxygenation; SOFA, sequential organ failure assessment.

### Subgroup analysis

**Figure 6:**
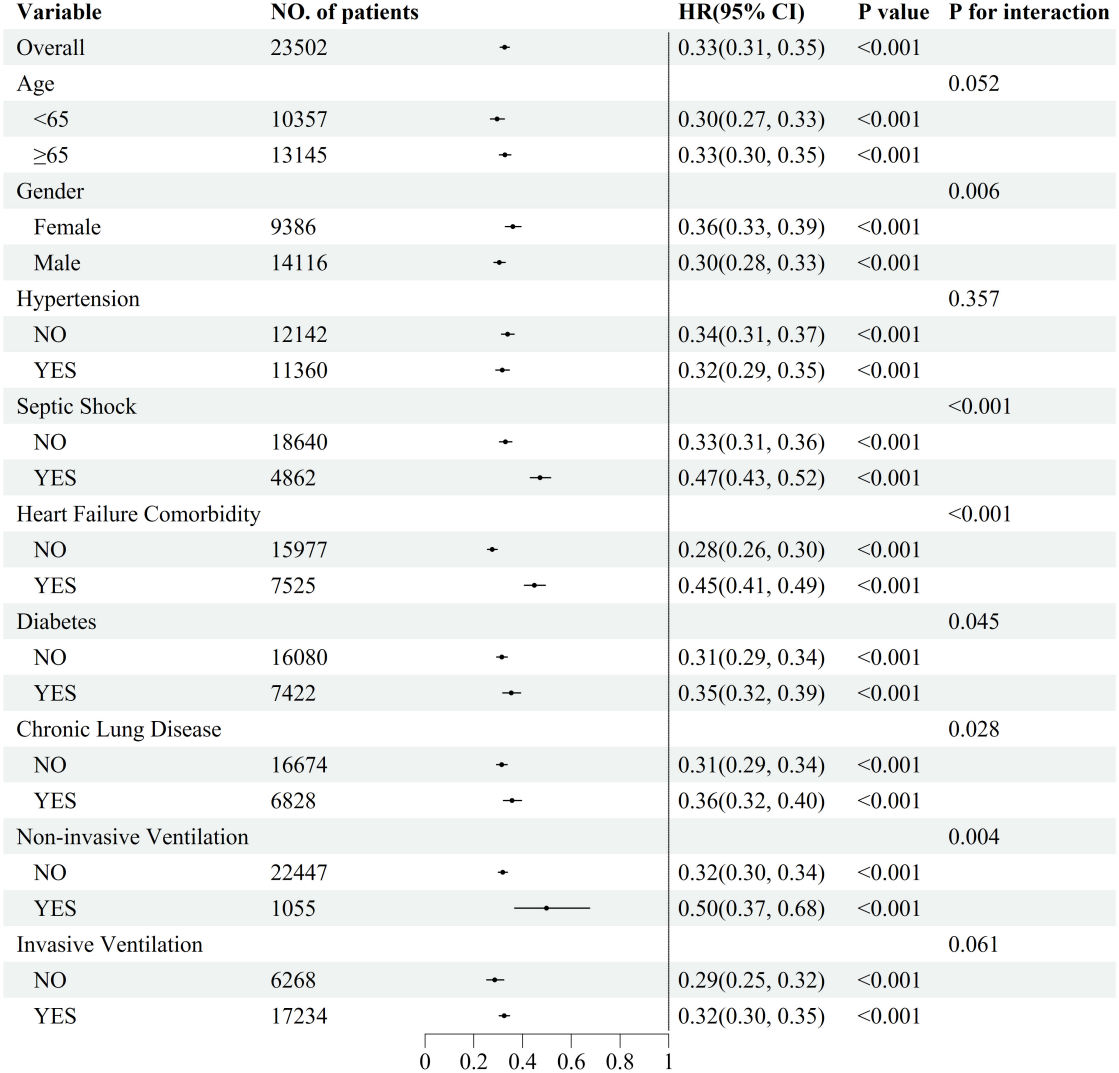
Subgroup analysis of the relationship between the ROX index and mortality, HR (95% CI) were derived from Cox proportional hazards regression models.

Figure 6 illustrates the association between the ROX index and 28-day mortality across different subgroups of sepsis patients. A higher ROX index demonstrated a consistent protective association against mortality in all subgroups (all *P* < 0.001). However, significant interaction effects were observed, indicating that the protective effect was significantly attenuated in high-risk subgroups, including patients with female, septic shock, heart failure, diabetes, chronic lung disease and non-invasive ventilation (*P* for interaction < 0.05 for all).

### Sensitivity analysis

To evaluate the robustness of our study findings, we conducted five sensitivity analyses. Firstly, we treated the ROX index as a continuous variable and adjusted for various confounding factors; the results were consistent with our primary findings (Supplementary Table S1). Secondly, we used the median value of the ROX index (9.67) as the cutoff for dichotomizing risk. Again, the results were consistent with our primary findings (Supplementary Table S1). Thirdly, considering that missing data could potentially diminish statistical power, we excluding all observations with any missing values. The results of this analysis were consistent with our primary outcomes (Supplementary Table S2). Fourthly, given that patients with concurrent pulmonary infections might exhibit lower ROX index values, we performed an analysis excluding such patients. The resultant findings remained in alignment with our principal outcomes (Supplementary Table S2). Lastly, to further address potential confounders, we included all covariates that were significant in the univariate analyses into a multivariate Cox regression model. This analysis showed that the model maintained its statistical significance, and the results were in line with the primary outcomes of this study (Supplementary Table S3).

## Discussion

In this study, we aimed to investigate the effect of the ROX index on sepsis patients. We found that the ROX index has a significant effect on the risk of death among sepsis patients; a reduction in the ROX index indicates an increased risk of mortality in these patients, even after adjusting for potential confounding variables. As the ROX index decreases, both the length of hospital stay and ICU length of stay increase, demonstrating a non-linear, “L”-shaped relationship. Specifically, when the ROX index reaches 10.50, 9.29, and 9.56 respectively, there is no longer a notable increase in mortality risk, hospital stay, or ICU length of stay with higher ROX index values. Additionally, the ROX index demonstrates significant predictive value for mortality in sepsis patients, suggesting that it can be used as a predictive tool in sepsis patients. In subgroup analysis, although stratification revealed variations in predicting mortality risk across different populations, overall, patients with a lower ROX index exhibited a higher risk of death compared to those with a higher index. Therefore, we believe that the ROX index holds considerable value for assessing the prognosis of sepsis patients and can be a useful tool for evaluating their outcomes.

In previous studies, the ROX index has been primarily used to identify patients with a higher risk of HFNC failure (6, 12, 13). It exhibits high sensitivity and specificity in predicting progression from HFNC to invasive mechanical ventilation (14). Given its simplicity, repeatability, non-invasiveness, and effectiveness in predicting respiratory failure outcomes (15), numerous studies have applied the ROX index for forecasting adverse outcomes in related diseases (13, 16, 17), and have demonstrated favorable predictive performance. Ahn et al. (18) conducted a comparative analysis between the ROX index and the incidence of mechanical ventilation and sepsis among Emergency Department patients with sepsis, revealing that the ROX index correlates with 24-h mechanical ventilation and short-term mortality in sepsis patients. Patients with a ROX index less than 5.238 exhibited a higher 28-day mortality rate. The determination of a universally applicable ROX index cut-off value remains challenging, in this study, we used survival analysis methods to establish a potential, data-derived ROX index threshold of 6.46 and found that patients with ROX values below this exploratory threshold faced a higher mortality risk. It is important to note that this threshold has not been externally validated and should be considered exploratory. Although there is no universal agreement on the cut-off value of the ROX index, the extent of these variations appeared to be somewhat limited (19).

Clinically, the relationship between biomarkers, prognostic indicators, and sepsis prediction is often nonlinear (20–22). Categorizing prognostic indicators may not fully show their link to disease outcomes and could misinterpret results. Thus, we used RCS curves to study this relationship and find the ROX index shows an L sheep relationship with the mortality risk, LOS, and ICU LOS in sepsis patients, when the ROX index reaches 10.50, further increases do not significantly raise mortality risk. Therefore, we believe that when the ROX index exceeds 10.50, its predictive value for the mortality risk in septic patients is limited. Similarly, when the ROX index reaches 9.29 and 9.56, further increases have limited effect on total LOS and ICU LOS.

Although many indicators are available to predict the death risk of sepsis patients, there is still no optimal indicator. New biomarkers for predicting the prognosis of sepsis are constantly being explored. However, their clinical applications remain unsatisfactory, with significant variations in clinical prediction outcomes and poor consistency. Heart rate, blood pressure, respiration, oxygen saturation, etc. are the most easily obtained clinical parameters in clinical practice and have some predictive value in predicting the prognosis of critically ill patients. However, their sensitivity and specificity are not very high. The SOFA score is not only one of the diagnostic criteria for sepsis, but also widely used to assess the prognosis and treatment of critically ill patients, including those with sepsis (23). However, the SOFA score relies on blood biochemistry related indicators, and its dynamic and continuous monitoring is limited. In contrast, the ROX index can be conveniently monitored dynamically and evaluated in real time. Therefore, we comparatively analyzed the efficacy of the ROX index and SOFA in assessing the prognosis of sepsis patients through diagnostic experiments. The results showed that the predictive efficacy of the SOFA score for mortality in sepsis patients was less than satisfactory. Although the AUC of the ROX index for prediction was also not very high, it was higher than that of the SOFA score, and its diagnostic ability was significantly better than that of the SOFA score. Therefore, we have reason to believe that the ROX index may be a more ideal indicator for predicting death in septic patients compared with the SOFA score.

As the ROX index is mainly calculated from SpO_2_, FiO_2_, and RR, and is primarily used to assess a patient's respiratory function (24), in septic patients, those with concurrent pneumonia may theoretically have a lower ROX index, which could affect the ROX index assessment of prognosis in septic patients. Therefore, in the sensitivity analysis, we considered the effect of missing values, cut-off values, and other factors on the results, after excluding all patients with concurrent lung infections, the results still showed that the ROX index had a predictive value for the risk of death in sepsis patients, which is consistent with the main results of this study. This finding is also consistent with the pathophysiological process of sepsis: during severe infections, regardless of whether the primary site of infection is the lung, under the stimulation of inflammatory mediators such as tumor necrosis factor-α (TNF-α) and interleukin-6 (IL-6) (25, 26), damage to the pulmonary capillary endothelium is induced, leading to an increase in alveolar-capillary permeability (27), triggering non-cardiogenic pulmonary oedema (28), an increase in respiratory rate, resulting in a decrease in SpO_2_, an increase in FiO_2_ and a consequent decrease in the ROX index. This process reflects the secondary effect of the systemic inflammatory response on respiratory function. The greater this impact, the worse the patient's respiratory function and the higher the risk of death. Therefore, from the perspective of the pathophysiological process of sepsis, the ROX index is also one of the potential indicators for predicting the risk of death in sepsis. However, since the ROX index has only moderate discriminatory ability, its true value may lie in its potential as a quick complement to more complex assessment tools, such as the SOFA score. This could help prioritize patients who may require closer monitoring and more aggressive intervention, especially in resource-limited settings.

However, this study had several limitations: firstly, it is a retrospective study using data only from the MIMIC-IV database with a long time span, which may introduce heterogeneity and limit the generalizability of the findings. Secondly, in this study, the ROX index was calculated as the average value with different indicators on the first day after the patient's admission. The effect of the dynamic changes in the ROX index on the outcomes of septic patients was not statistically analyzed. Additionally, the ROX values were measured at varying time points relative to sepsis onset, which may introduce selection bias, limiting the extrapolation and generalization of the findings. Thirdly, although we have controlled for as many confounding factors as possible, the limitations inherent to this retrospective study mean that there may still be numerous potential unmeasured confounders. This study did not fully consider the effect of mechanical ventilation status on the ROX index. The treatment parameters of mechanically ventilated patients may affect the strength of the association between the ROX index and true lung function status, leading to a deviation in the predictive efficacy of this index in this population. Therefore, while our findings demonstrate an association between the ROX index and mortality in sepsis patients, establishing causality remains challenging due to the potential for unmeasured confounding. Moreover, this study lacks multicenter external validation. The current conclusions are mainly based on a single database. Although this database has undergone rigorous quality control, differences in data collection may limit the universality of the research findings. Although this study adjusted for many confounding factors, given the complexity of sepsis and differences in treatment between patients, there may still be many unadjusted confounding factors, which may lead to residual confounding bias. Finally, this study did not investigate whether combining the ROX index with other established scores such as SOFA or APACHE II could yield a composite score with superior predictive accuracy, which represents an important avenue for future research.

The results of this study have the following advantages. First, there are few studies on the correlation between the ROX index and sepsis. This study further evaluates the correlation between the ROX index and the prognosis of sepsis, providing a reference for the clinical application of the ROX index in sepsis. Second, this study explores the correlation between the ROX index and sepsis from multiple perspectives and conducts sensitivity analyses. Third, through RCS curves, this study quantifies the associations between the ROX index and prognosis, LOS, and ICU LOS in septic patients. Fourth, the ROX index, the main indicator explored in this study, is easy to calculate, non-invasive, and can be easily implemented and dynamically monitored at the bedside, thus providing a potential marker for the evaluation of sepsis prognosis.

## Conclusions

In summary, our study reveals that in patients with sepsis, a higher ROX index is associated with lower mortality rates and shorter LOS and ICU LOS. However, the ROX index demonstrates moderate predictive accuracy but outperforms the SOFA score. Further prospective studies are needed to confirm these results and evaluate the ability of the ROX index to predict outcomes in sepsis patients.

## Data Availability

Publicly available datasets were analyzed in this study. This data can be found at: https://physionet.org/content/mimiciv/3.0. Data are, however, available from the authors upon reasonable request and with permission from the Massachusetts Institute of Technology (MIT) and Beth Israel Deaconess Medical Center (BIDMC). Interested parties should contact the corresponding author to request access to the data.
