# Supplementary material for: Association of the ROX index with mortality in sepsis patients: a retrospective study
Source: Front Med (Lausanne). 2025 Dec 2;12:1709669. doi: 10.3389/fmed.2025.1709669 (PMC12705608; doi:10.3389/fmed.2025.1709669)
Supplement: Supplementary file 1 [file Table_1.docx]

Supplementary Material

# Supplementary Tables

**Supplementary Table 1.** Sensitivity analysis of cox regression models using ROX index with different variable types

| **Exposure** | **Crude model** | | **Model 1** | | **Model 2** | | **Model 3** | |
| --- | --- | --- | --- | --- | --- | --- | --- | --- |
|  | **HR (95% CI)** | ***P* value** | **HR (95% CI)** | ***P* value** | **HR (95% CI)** | ***P* value** | **HR (95% CI)** | ***P* value** |
| Continuous^a^ |  |  |  |  |  |  |  |  |
| ICU- Mortality | 0.87(0.86-0.88) | <0.001 | 0.87(0.86-0.88) | <0.001 | 0.87(0.86-0.88) | <0.001 | 0.87(0.86-0.88) | <0.001 |
| 28-day Mortality | 0.87(0.87-0.88) | <0.001 | 0.87(0.86-0.88) | <0.001 | 0.87(0.86-0.88) | <0.001 | 0.88(0.87-0.88) | <0.001 |
| 14-day Mortality | 0.86(0.85-0.86) | <0.001 | 0.85(0.84-0.86) | <0.001 | 0.85(0.84-0.86) | <0.001 | 0.86(0.85-0.87) | <0.001 |
| 7-day Mortality | 0.82(0.81-0.83) | <0.001 | 0.81(0.80-0.82) | <0.001 | 0.81(0.80-0.82) | <0.001 | 0.82(0.81-0.83) | <0.001 |
| Median^b^ |  |  |  |  |  |  |  |  |
| ICU- Mortality | 0.50(0.46-0.53) | <0.001 | 0.48(0.45-0.52) | <0.001 | 0.48(0.44-0.52) | <0.001 | 0.50(0.47-0.54) | <0.001 |
| 28-day Mortality | 0.52(0.50-0.56) | <0.001 | 0.51(0.49-0.54) | <0.001 | 0.51(0.48-0.54) | <0.001 | 0.54(0.51-0.57) | <0.001 |
| 14-day Mortality | 0.48(0.45-0.52) | <0.001 | 0.47(0.44-0.51) | <0.001 | 0.47(0.44-0.50) | <0.001 | 0.50(0.47-0.53) | <0.001 |
| 7-day Mortality | 0.39(0.36-0.43) | <0.001 | 0.39(0.36-0.42) | <0.001 | 0.39(0.35-0.42) | <0.001 | 0.41(0.38-0.45) | <0.001 |

Crude model adjusted for nothing.

Model 1 adjusted for age.

Model 2 added gender to Model 1.

Model 3 included SOFA score in addition to Model 2.

^a^ Including the ROX index as a continuous variable.

^b^ Using the median ROX index value of 9.67 as the cutoff, the Cox model was then constructed.

**Supplementary Table 2.** Sensitivity analysis of cox regression models using different datasets

| **Exposure** | **Crude model** | | **Model 1** | | **Model 2** | | **Model 3** | |
| --- | --- | --- | --- | --- | --- | --- | --- | --- |
|  | **HR (95% CI)** | ***P* value** | **HR (95% CI)** | ***P* value** | **HR (95% CI)** | ***P* value** | **HR (95% CI)** | ***P* value** |
| Without missing values ^a^ |  |  |  |  |  |  |  |  |
| ICU- Mortality | 0.40(0.37-0.43) | <0.001 | 0.38(0.35-0.41) | <0.001 | 0.38(0.35-0.41) | <0.001 | 0.40(0.37-0.43) | <0.001 |
| 28-day Mortality | 0.35(0.33-0.37) | <0.001 | 0.33(0.31-0.35) | <0.001 | 0.33(0.31-0.35) | <0.001 | 0.35(0.33-0.37) | <0.001 |
| 14-day Mortality | 0.32(0.30-0.34) | <0.001 | 0.31(0.28-0.33) | <0.001 | 0.31(0.28-0.33) | <0.001 | 0.32(0.30-0.34) | <0.001 |
| 7-day Mortality | 0.27(0.25-0.30) | <0.001 | 0.26(0.24-0.29) | <0.001 | 0.26(0.24-0.29) | <0.001 | 0.28(0.26-0.30) | <0.001 |
| Without pulmonary infections ^b^ |  |  |  |  |  |  |  |  |
| ICU- Mortality | 0.28(0.25-0.31) | <0.001 | 0.27(0.25-0.30) | <0.001 | 0.27(0.25-0.30) | <0.001 | 0.29(0.26-0.32) | <0.001 |
| 28-day Mortality | 0.23(0.22-0.25) | <0.001 | 0.23(0.21-0.25) | <0.001 | 0.23(0.21-0.25) | <0.001 | 0.25(0.23-0.27) | <0.001 |
| 14-day Mortality | 0.22(0.20-0.24) | <0.001 | 0.21(0.20-0.23) | <0.001 | 0.22(0.20-0.23) | <0.001 | 0.23(0.21-0.25) | <0.001 |
| 7-day Mortality | 0.19(0.18-0.21) | <0.001 | 0.19(0.18-0.21) | <0.001 | 0.19(0.18-0.21) | <0.001 | 0.21(0.19-0.23) | <0.001 |

Crude model adjusted for nothing.

Model 1 adjusted for age.

Model 2 added gender to Model 1.

Model 3 included SOFA score in addition to Model 2.

^a^ Excluding patients with missing values.

^b^ Excluding patients with pulmonary infections.

**Supplementary Table 3.** Univariate and Multivariate Cox Regression Analyses of the ROX Index

| **Variable** | **Univariate analysis** | | **Multivariable analysis** | |
| --- | --- | --- | --- | --- |
|  | **HR (95% CI)** | **p value** | **HR (95% CI)** | **p value** |
| Age | 1.02(1.02-1.03) | <0.001 |  |  |
| Weight | 0.99(0.99-0.99) | <0.001 | 0.99(0.99-0.99) | <0.001 |
| Male | 0.88(0.83-0.92) | <0.001 | 0.93(0.87-0.98) | 0.010 |
| Hypertension | 0.82(0.78-0.86) | <0.001 | 0.93(0.88-0.98) | 0.010 |
| Septic Shock | 2.89(2.73-3.05) | <0.001 | 1.47(1.38-1.56) | <0.001 |
| Heart Failure Comorbidity | 1.33(1.26-1.41) | <0.001 | 0.80(0.75-0.85) | <0.001 |
| Diabetes | 1.01(0.95-1.07) | 0.846 |  |  |
| Chronic Lung Disease | 1.10(1.04-1.17) | 0.001 | 0.95(0.90-1.01) | 0.138 |
| CCI | 1.17(1.16-1.18) | <0.001 | 1.09(1.07-1.10) | <0.001 |
| SOFA Score | 1.14(1.13-1.15) | <0.001 | 1.01(1.00-1.02) | 0.168 |
| SAPS II Score | 1.05(1.05-1.05) | <0.001 | 1.02(1.02-1.03) | <0.001 |
| HFNC | 1.25(1.13-1.38) | <0.001 | 0.94(0.85-1.04) | 0.228 |
| Non-invasive Ventilation | 0.75(0.65-0.87) | <0.001 | 0.75(0.65-0.87) | <0.001 |
| Invasive Ventilation | 1.51(1.41-1.61) | <0.001 | 1.17(1.08-1.25) | <0.001 |
| Heart Rate | 1.01(1.01-1.02) | <0.001 | 1.00(1.00-1.00) | <0.001 |
| Respiratory Rate | 1.04(1.04-1.04) | <0.001 | 1.00(1.00-1.01) | 0.185 |
| Mean blood pressure | 0.98(0.97-0.98) | <0.001 | 0.99(0.99-1.00) | <0.001 |
| ROX Index | 0.87(0.87-0.88) | <0.001 | 0.92(0.91-0.93) | <0.001 |
| WBC count | 1.01(1.01-1.01) | <0.001 | 1.00(1.00-1.00) | 0.881 |
| Hemoglobin | 0.98(0.97-0.99) | 0.001 | 1.06(1.05-1.08) | <0.001 |
| Platelet count | 1.00(1.00-1.00) | 0.195 |  |  |
| BUN | 1.01(1.01-1.01) | <0.001 | 1.00(1.00-1.01) | <0.001 |
| Creatinine | 1.06(1.05-1.06) | <0.001 | 0.97(0.95-0.99) | 0.001 |
| Potassium | 1.21(1.19-1.23) | <0.001 | 1.02(0.99-1.05) | 0.246 |
| Sodium | 1.03(1.02-1.03) | <0.001 | 1.05(1.04-1.05) | <0.001 |
| Chloride | 0.98(0.97-0.98) | <0.001 | 0.96(0.96-0.97) | <0.001 |
| Calcium | 1.15(1.12-1.18) | <0.001 | 1.01(0.98-1.03) | 0.609 |
| PT | 1.02(1.02-1.02) | <0.001 | 1.01(1.00-1.01) | <0.001 |
| INR | 1.18(1.16-1.19) | <0.001 | 1.02(0.98-1.05) | 0.404 |
| Lactate | 1.07(1.07-1.07) | <0.001 | 1.05(1.05-1.06) | <0.001 |

*CCI* Charlson comorbidity index, *SOFA* sequential organ failure assessment, *SAPS II* Simplified acute physiology score II, *HFNC* High-Flow Nasal Cannula, *ROX* Respiratory Oxygenation, *WBC* White Blood Cell, *BUN* Blood Urea Nitrogen, *PT* Prothrombin Time, *INR* International Normalized Ratio
